# Family medicine residents’ risk of adverse motor vehicle events: a comparison between rural and urban placements

**Published:** 2013-09-30

**Authors:** Fred Janke, Bonnie Dobbs, Rhianne McKay, Meghan Linsdell, Oksana Babenko

**Affiliations:** 1 Dept. of Family Medicine, University of Alberta, Edmonton, Alberta Canada; 2 Medically At-Risk Driver Centre, University of Alberta, Edmonton, Alberta, Canada

## Abstract

**Background:**

Sleep deprivation and fatigue are associated with long and irregular work hours. These work patterns are common to medical residents. Motor vehicle crashes (MVCs) are a leading cause of injury related deaths in Canada, with MVC fatality rates in rural areas up to three times higher than in urban areas.

**Objectives:**

To: 1) examine the number of adverse motor vehicle events (AMVEs) in family medicine residents in Canada; 2) assess whether residents with rural placements are at greater risk of experiencing AMVEs than urban residents; and 3) determine if family medicine residency programs across Canada have travel policies in place.

**Methodology:**

A prospective, cross-sectional study, using a national survey of second-year family medicine residents.

**Results:**

A higher percentage of rural residents reported AMVEs than urban residents. The trend was for rural residents to be involved in more MVCs during residency, while urban residents were more likely to be involved in close calls. The majority of Canadian medical schools do not have resident travel policies in place.

**Conclusion:**

AMVEs are common in family medicine residents, with a trend for the number of MVCs to be greater for rural residents. These data support the need for development and incorporation of travel policies by medical schools.

## Introduction

Motor vehicle crashes (MVCs) are a leading cause of injury related deaths in Canada.^1^ In addition to factors such as amount driven, speed, and seat belt use, driver fatigue and/or sleepiness are known risk factors for adverse motor vehicle events (AMVE), including crashes.^2^–^5^ Medical residents may be especially susceptible to MVCs after a night on call due to lack of sleep.^2^,^5^ In a survey by Steier and colleagues,^6^ physicians who had been in a MVC within the previous year reported that they remembered being sleepy immediately before the MVC. Forty-nine percent of physicians in another study^3^ reported that they had fallen asleep at the wheel of their vehicle, with 90% of these events occurring after a night on call. In a study of emergency medicine residents, Steele et al.^7^ found that 74% of MVCs and 80% of near-crashes were reported after working a night shift compared to after working day and evening shifts.

In a number of studies, the effects of sleep deprivation on performance have been compared to alcohol intoxication.^8^–^11^ For example, in an examination of male drivers aged 20–50 years, Fairclough and Graham^12^ found that sleep-deprived participants made critical safety errors (i.e., errors that would be likely to cause a crash, such as lane crossing), similar to individuals with a blood alcohol content (BAC) of between 0.08–0.10% during simulated driving performance. Arendt and colleagues^13^ compared the neurobehavioral performance of medical residents during a heavy call rotation (80–90 hours per week with night call every fourth night) to those during a light call rotation (44 hours per week, with night call only if the on-call resident became ill). Results indicated that the neurobehavioral performance of residents after a heavy call rotation was similar to that of residents with a BAC of 0.04–0.05% after a light call rotation, suggesting that the judgment and thinking abilities are impaired by sleep deprivation as much as by alcohol consumption.

Fatigue is not the only factor that can affect MVC risk. There have been several studies that have shown that MVC risk^14^,^15^ and fatalities^14^–^16^ are significantly higher in rural than in urban areas,^14^–^16^ with the pattern of findings consistent whether using national data,^17^ province-specific data,^15^ or data from special populations.^15^,^17^ Zwerling and colleagues^14^ found that fatal MVC incidence density (number of fatal crashes/number of miles per 100 million miles driven) in the United States was 2.86 in rural areas compared to 1.28 in urban areas, and the crash injury rate (number of crashes with an injury/number of all crashes per 1,000 crashes) was 372.25 in rural areas compared to 331.55 for those in urban areas. Zwerling offered several explanations for these differences including the increased severity of MVCs in rural versus urban areas, a higher preponderance of people not wearing seat belts in rural areas compared to those in urban areas, and increased medical transport time to care in rural areas. Kmet and Macarthur^15^ found similar results in a study of fatal MVCs in Alberta, Canada. In that study, the fatal MVC rate per 100,000 individuals was 11.3 in rural areas, a rate that was nearly six times higher than in urban areas of the province (1.9 per 100,000 individuals). Anecdotal evidence from the Director of the Rural Medicine Program at the University of Alberta in Edmonton, Canada, suggests that medical residents completing their rural rotation may be at higher risk of AMVEs than their urban counterparts. If true, the findings have important safety implications for residents completing rural residency rotations. As such, information on the risks of driving while sleep deprived could be incorporated into medical residency training programs and policies could be developed to minimize medical residents driving while sleepy in both rural and urban residency rotations.

The purpose of this study was threefold: 1) to examine the number of AMVEs in family medicine residents in Canada; 2) to assess whether family medicine residents with rural placements are at greater risk of experiencing AMVEs than their urban counterparts; and 3) to determine if family medicine residency programs across Canada have policies in place related to resident travel during residency.

## Methods

### Adverse Motor Vehicle Events (AMVEs)

Survey methodology was used for data collection relative to the first two objectives. The target sample was second year residents (PGY2) in family medicine in Canada. Since the survey inquired about 22 months of driving experience, only PGY2 residents would have accumulated this length of driving time within their residency and thus were the only ones included. Based on Canadian Resident Matching Services (CaRMS) 2008 data, there were approximately 1,049 PGY1 family medicine residency positions available across Canada, a number that corresponds approximately to the number of residents who would be in the second year of their post graduate program one year later (PGY2).

Recruitment of PGY2 residents in family medicine was done through their respective departments of family medicine in universities across Canada. The program secretary of each residency program acted as the primary contact for residents within her/his respective program. Documents outlining the purpose of the study and the study procedure were provided to each program secretary. The program secretary was also provided with documents for the residents, with a request to forward the documents to each of the PGY2 residents. That contact occurred during the last two months of their residency. Residents were asked to provide informed consent and complete a structured, web-based questionnaire by following an electronic link that was sent in the email. To ensure anonymity, the survey software automatically assigned each resident a computer-generated study identification number. A reminder notice was sent out to all residents after two weeks, asking those individuals who had not completed the questionnaire to do so. This process was repeated after a further 2 weeks.

The structured questionnaire, designed by the study team, consisted of demographic questions (age, gender, etc.); type of program (rural vs. urban); residency location (province); driving habits (e.g., estimated number of kilometres driven); AMVEs (number of crashes, number of injury producing crashes, citations, other adverse events such as close calls, etc.); and type and nature of injuries (where applicable) during the first 22 months of the residency program (i.e., July 1, 2007 to April 30, 2009 *–* hereafter referred to as the ‘study period’).

### Policies related to family medicine resident travel during residency

To determine if family medicine residency programs across Canada have residency travel policies in place, the following steps were taken. First, a search on the website of each school that has a family medicine program was performed. Second, if a travel policy could not be found through a search, each school was contacted and asked whether a travel policy was in place, and if so, the travel policy document was requested from that school (see Appendix A for an example from one school). The travel policies were examined in terms of their content and level of detail.

Ethical approval for both phases of the research was obtained from the University of Alberta’s Health Research Ethics Board (Panel B) and from each of the individual medical schools when its own institutional approval was required.

## Data Analyses

### Adverse Motor Vehicle Events

Descriptive statistics (means, standard deviations, frequencies) were used to describe the sample, with t-tests/ANOVAs (as appropriate) and chi-square analyses used to test for differences between groups on demographic and primary outcome measures. A liberal *alpha* value of 0.10 was chosen given theexploratory nature of the study and the fact that aType II error (i.e., saying there is no difference between the rural and urban residents on the AMV measures when in fact there is) was of a more important concern in the present study, with a potential to inform driving policy in medical schools. Increasing alpha from 0.05 to 0.10 decreases the chances of making a Type II error.^18^ The data were analyzed in aggregate form (overall and urban vs. rural) to ensure participant anonymity. Due to low response rates in some provinces, data analyses by province were not performed.

### Policy related to family medicine resident travel during residency

Descriptive statistics (frequencies) were used to analyze the data related to family medicine resident travel policy.

## Results

### Adverse Motor Vehicle Events

#### Sample as a whole

One hundred and forty-one PGY2 residents completed the survey. From a national perspective, percentages of residents who completed the survey were the highest in Alberta and Manitoba (67% and 45%, respectively), whereas the lowest percentages of residents were observed in Quebec and Saskatchewan (16% and 11%, respectively) (Table 1).

Overall, the mean age of the sample was 32.13 (*SD* = 5.39), with a range of 25*–*52 years (Table 2). A higher percentage of females (*n* = 93 or 66% of the sample) completed the survey. In terms of residency location, a higher percentage of urban residents completed the survey (79% urban vs. 21% rural). The average years of driving was 13.73 years (*SD* = 5.32). The average number of kilometres driven in the first 22 months of residency was 25,613 (*SD* = 19,436), with an average of 16,010 (*SD* = 15,774) kilometres driven for residency purposes.

Thirty-one (22%) of the residents reported having MVCs during the first 22 months of residency, with 26 residents reporting 1 crash, 3 residents reporting 2 crashes, one resident reporting 3 crashes, and one resident reporting 4 crashes, for a total of 39 crashes. Approximately two-thirds (61%) of the crashes were related to work, with the majority (36%) of work-related crashes having occurred ‘after work’ (defined as ‘on the drive home from work’). Two residents reported injuries as a result of the MVC, with one of the injuries requiring medical attention.

Seventy-six residents (54%) reported having a close call (defined as the potential for a crash but managing to avoid the adverse event). Eighteen residents reported having one close call during the first 22 months of residency, 18 residents reported having 2 close calls, 18 residents reported having 3 close calls, 7 residents reported having 4 close calls, and 14 residents reported having 5 close calls for a total of 206 close calls reported during the study period. Fifty-three (38%) residents reported receiving a citation (excluding parking tickets) during the first 22 months of residency.

Residents were asked the reason for their AMVE. Thirty-four percent cited fatigue, 25% cited driver inattention, 24% cited the weather, 12% cited road conditions, and 5% cited a wildlife encounter as the reason for the AMVE.

#### Rural vs. Urban

The mean age of both rural and urban respondents was 32 years. For both locations, a higher percentage of females responded to the survey (68% urban and 59% rural). The difference in response rate as a function of gender and location was not statistically significant (*p* = 0.35). Rural residents had driven fewer years on average (12.48 years vs. 14.05 years for urban residents), but the difference was not statistically significant (*p* = 0.16). Total kilometres driven during the first 22 months of residency and kilometres driven specifically for residency purposes differed significantly between the two groups, with rural residents driving on average a greater number of kilometres (37,103 vs. 22,528, respectively) (*p* < 0.001) and more than double the number of kilometres on average for residency purposes (28,804 vs. 12,663) (*p* < 0.001).

To determine if there were differences in AMVEs as a function of residency placement, we examined rates of MVCs and close calls between rural and urban residents. Overall, 20 of the 112 urban residents (17.9%) had a total of 26 crashes (Mean = 0.23 crashes/resident) compared to 11 of the 29 (37.9%) rural residents who had a total of 13 crashes (Mean = 0.45 crashes/resident) (Table 2). That is, the number of residents in MVCs as well as the number of MVCs per resident were both significantly higher for rural than for urban residents (*p* = 0.03 and *p* = 0.09, respectively). However, when adjusted for exposure (number of crashes/100,000 kilometres driven), the mean difference in the number of MVCs between urban and rural residents, irrespective of time of MVCs, was not statistically significant (1.18 vs. 1.99, respectively) (*p* = 0.27). Similarly, the mean difference in the number of work-related MVCs for the two groups of residents when adjusted for exposure (Number of crashes/100,000 kilometres driven) was not statistically significant (1.22 vs. 1.87, urban vs. rural, respectively) (*p* = 0.47) (Table 3). As noted previously, two of the MVCs resulted in injury (one urban and one rural resident), with the rural resident’s crash resulting in the need for medical attention (Table 2).

Fifty-seven of the 112 urban residents (50.9%) had a total of 157 close calls (Mean = 1.40 close calls/resident) compared to 19 of the 29 rural residents (65.5%) who had a total of 49 close calls (Mean = 1.69 close calls/resident) (Table 2). However, the differences in the percentages of residents involved in close calls as well as the number of close calls per resident for the two groups of residents were both determined not significant (*p* = 0.21 and *p* = 0.42, respectively). The average of work-related close calls when adjusted for exposure (number of close calls/10,000 kilometres driven) was higher for urban residents (2.74) than for rural residents (0.62), though this was not statistically significant (*p* = 0.29) (Table 3). Urban residents reported more close calls after work (60%) while rural residents reported that most of their close calls were unrelated to work (45%). However, rural residents still experienced a high rate of close calls after work (35%). Finally, a greater percentage of rural residents reported having received a citation (45%) versus 36% for urban residents, although the difference was not statistically significant (*p =* 0.39) (Table 2).

Residents were also asked what they thought was the potential cause of their AMVE (driver inattention, fatigue, weather, wildlife, road conditions, or other). A higher percentage of urban residents identified fatigue as the cause for the AMVE (40%) followed by inattention (27%) and weather conditions (24%), while rural residents were most likely to blame the weather (23%), fatigue (21%), and inattention (21%) as causes of AMVEs. These differences were not statistically significant. Not surprisingly, compared to urban residents, rural residents were more likely to identify ‘wild animals on the road’ as the cause of an AMVE (19% vs. 0%) (*p* < 0.001). Rural residents also were more likely than urban residents to identify [poor] road conditions as the cause of an AMVE (17% vs. 9%, respectively) (*p* = 0.03).

Finally, given a somewhat lower than expected response rate and the fact that the observed mean differences for the four outcome variables were determined not to be statistically significant (see Table 3), we performed power analysis in an attempt to explain non-significant results. However, due to the lack of research in this area (i.e., AMV events among medical residents), it was difficult to make any hypothesis with respect to the effect sizes to be expected. At the same time, we had no grounds to expect high effect sizes, and thus, the power analysis was based on the observed effect sizes and the liberal alpha level of 0.10. Increasing the alpha level from 0.05 to 0.10 increases statistical power because the null hypothesis (i.e., no difference) will be rejected more often, and consequently, the true alternative hypothesis (i.e., there is a difference) will have a greater chance of being accepted (i.e., power).^18^ The observed average effect size (Cohen’s *d*) for the four outcome variables was 0.20 (Table 3). Based on this effect size, and with a power of at least 80%, 310 participants would be required for each group to obtain significant results at the chosen *alpha* level of 0.10. This could potentially have been achieved with a higher response rate and, most importantly, the full participation of medical schools in the present study. If all the medical schools in Canada had participated in the study, the total number of family residents available for surveying would have been 1049, and depending on how each medical school defines their rural and urban residencies, in total between 250 and 350 rural residents could have been expected during our survey period.

### Policy related to family medicine resident travel during residency

To determine if family medicine residency programs across Canada have policies in place related to resident travel during residency, a national survey was conducted, with all 14 English speaking schools contacted by email. Follow-up phone calls were made to schools not responding to emails. Of the 14 schools that were sent emails or follow-up phone calls, 12 responded representing an 86% response rate. The majority of the responding schools (7, or 58%) did not have a policy in place as of August, 2011 (Table 4).

## Discussion

In our national survey of second year family medicine residents, the risk of AMVEs overall was high for both urban and rural residents, with rural residents significantly more likely to be involved in a MVC. There were also significantly more MVCs per rural resident than urban. After adjusting for exposure, rural residents also had a higher number of crashes and number of work related crashes per 100,000 kilometres driven, but those differences failed to reach statistical significance. Finally, urban residents reported more close calls when adjusted for exposure compared to rural residents.

The observed trend for rural residents to be involved in more MVCs and for urban residents to be involved in more close calls, when adjusted for exposure, is unexpected. The design of rural roads (e.g., narrow, more curves, faded markings, etc.) may be less safe than urban roads, potentially leading to higher accident rates.^19^ Conversely, higher vehicle volume and increased prevalence of other road users in urban locations (e.g., pedestrians, cyclists) may account for the higher number of close calls reported by urban residents. When compared to other studies of medical residents, a higher percentage of residents in the current study experienced MVCs (22% vs. 8%,^20^ 8%,^7^ and 13%^6^) with more MVCs per resident in the current study (0.28 vs. 0.14^2^). Methodological differences may account for these findings.

Landrigan and colleagues,^20^ using prospective methodology, had pediatric residents from three large pediatric training programs in Boston, Stanford, and Washington complete daily logs on hours worked, hours of sleep, as well as MVCs and near misses. The data were collected the spring before and after the introduction of work hour limits for residents. It may be that the completion of daily logs documenting the number of hours worked and amount of sleep heighted the residents’ awareness of the effects of fatigue on routine activities such as driving, resulting in a modification of behaviour. A shorter study time (12 months vs. 22 months for the current investigation) also helps to explain the differences in MVC rate between the two studies. Prospective methodology was also used in the study of first year residents conducted by Barger and colleagues.^2^ Although survey methods (web-based survey) were similar to that used in the current study, residents reporting a MVC in the Barger et al. study were requested to provide documentation of the crash (e.g., police report, insurance claim, automobile repair record, medical record, photograph of the damaged vehicle, or a written description of the crash), a request that may have resulted in the under-reporting of crashes. In addition, residents in the Barger et al. study were asked to complete a monthly survey. Of the 2,737 participants completing the baseline survey, only 682 completed all 12 surveys, with the remaining 1,550 participants completing 2 or more monthly surveys. Thus, a significant number of residents completed fewer than 12 months of surveys. As a result, the differences in study time for a MVC to occur across residents differed significantly from the current investigation. In the Steele et al.^7^ study, surveys were distributed by mail to Emergency Medicine residents in the United States, with 1,554 usable surveys returned. As noted above, the reported rate of MVCs in that study was lower than found in the present investigation (22% vs. 8%). However, the definition of MVC in the Steele et al. study was limited to a crash occurring while driving home from an emergency department shift, whereas our definition included having had a crash at any time during the first 22 months of residency. Notably, the time frame for data collection (e.g., number of months) was not reported by Steele and colleagues. The limited time frame (e.g., driving home) for a MVC in the Steele et al. study, as well as potential differences in time periods between the Steele et al. and the current study, may account for differences in the rate of MVCs between the two studies. In the study by Steier and colleagues^6^, 38 physicians, 37 nurses, and 40 hi-tech workers were asked to self-report MVCs in the last year. The overall reported crash rate (13%) was for the sample as a whole. It is reasonable to assume that nurses and hi-tech workers may not be as ‘at-risk’ for MVCs due to shorter and more regular work hours compared to their physician counterparts. Thus, the inclusion of two groups of participants with a lower risk of MVCs, as well as the shorter study period, may have resulted in a lower risk of MVCs than that found in the current investigation. Finally, none of the studies investigating medical residents’ risk of MVCs have stratified their sample by urban and rural placement.

Interestingly, in our study, a higher percentage of AMVEs were ‘unrelated to work’ for rural residents. It may be that the nature of rural residency is such that the need for travel and distances travelled for ‘after work’ activities (e.g., shopping, entertainment, etc.) are greater for rural residents than for urban residents. It is also the case that travel that is ‘unrelated to work’ (e.g., travelling into urban centres on days off) is greater for rural residents than for their urban counterparts, increasing the opportunity for an AMVE. Irrespective of time of occurrence, the higher percentage of AMVEs for rural residents is cause for concern and needs to be explored in future research. Future research should also include residents at all schools with family medicine programs in order to increase the sample size. It also would be useful to examine AMVEs of residents before driving policies were implemented at schools and then after implementation in order to see if the changes in policies resulted in change.

Despite the documented relationship between sleepiness and AMVEs in medical residents, it appears that few medical schools offer advice on the role of sleepiness and driving. No published studies on this topic were found in Canadian schools. There is, however, published literature from the United Kingdom, with one study indicating that only 6 medical schools offered students advice on how to avoid MVCs.^21^ Our survey results from programs in Canada indicate that the majority of programs do not have travel policies in place for residents (Table 4). For those programs that do have policies, the policies vary in detail and the circumstances covered. Most of the policies state that residents should not drive in inclement weather and should not be on call prior to driving a long distance. At the Department of Family medicine at the University of Alberta, we have instituted a travel policy to account for safe driving conditions. The travel policy applies to the rural stream of the family medicine residency program (Appendix A). The travel policy applies to rural residents only, and not to urban residents, as it documents policy on driving long distances in poor weather conditions, an issue not concerning the urban program. For example, urban program residents are excused from mandatory academic activities if they are situated more than 50 kilometers from the city. Note, however, that in the rural program this distance is considerably farther at 350 kilometers which increases the amount of time rural residents spend on the road. Based on the results from our survey and a review of current practices regarding driving policies for residents, our recommendation would be for programs to include a formal policy related to residency travel within their departments.

It is of interest that recent guidelines regarding resident duty hours have been published by the Accreditation Council for Graduate Medical Education (ACGME) in the United States.^22^ The guidelines dictate the number of consecutive and weekly hours that residents are permitted to work as a means of managing the adverse effects of sleep deprivation. Specifically, in the ACGME guidelines, it states that residents can work no more than 80 hours per week averaged over a 4 week period and that all residents will be assigned a minimum of one day free of duty per week when averaged over 4 weeks. In addition, duty hours cannot exceed 16 hours for PGY1 residents and 24 hours for residents in PGY2 and above. The use of ‘strategic napping’ also is recommended for overnight shifts.^22^ Currently, there are no consensus guidelines for Canadian residents and restrictions can vary by province. For example, in Manitoba, residents are limited to working 89 hours per week,^23^ whereas in the Maritime Provinces, the limit for resident work is 90 hours per week^24^ (both averaged over a 4 week period). Residents in Quebec are limited to 78 hours of work per week over a 28-day rotation and no more than 16 hours per shift.^25^ Even with these guidelines, it may be that residents are working in excess of the recommended maximum, potentially increasing the danger to patients via medical errors and to themselves via increased crash risk following an extended work shift. Notably, the Canadian Association of Internes and Residents (CAIR) has recently released a position paper on resident duty hours. In that paper CAIR “calls on all PGME departments, employers, governments, and other relevant stakeholders to ensure…that physicians’ duty hours must be managed such that they do not in any way endanger their health or the health of patients”.^26^

It is important to acknowledge the limitations of our study. First, the data are based on self-report which has the potential to influence the accuracy of the data. Previous research on AMVEs using self-report indicates that the events often are underreported.^27^ Thus, the data presented here may actually under-represent the scope of the problem. On the other hand, sampling bias may account for our findings in that residents experiencing AMVEs may have been more likely to complete the survey. Unfortunately, we have no way of determining whether sampling bias was present in our research. Lastly, the overall response rate from residents, based on participating schools, was 34%, a response rate that is consistent with rates reported for web-surveys.^28^ The somewhat low response rate was in part dictated by the school’s policy of access to residents (Table 1). Despite the limitations, the strength of this research is that, to our knowledge, this study is the first attempt to survey, on a national level, the frequency of AMVEs for family medicine residents in Canada.

In order to decrease the probability that AMVEs occur, it is important to take driving safety into consideration when planning educational activities. This research has the potential to inform on policies related to safety issues for family medicine residency programs in both rural and urban locations across Canada. The research also helps to increase awareness of factors that may lead to AMVEs (e.g., fatigue, distraction, etc.), which in turn could lead to future enhancement of driving safety through behaviour change.

Finally, as family medicine program expansion incorporates more learners into the rural environment, driving safety and travel policies become a more important consideration. Driving safety may be a motivational force to explore other avenues for structured learning such as web-based interfaces and video-conferencing. The latter are not without disadvantages and require support from the respective Information Technology (IT) departments at each university. Sometimes IT departments are slow to meet these challenges. However, if there is a concern for resident safety, then there is reason to make the support of alternative methods for distributed learning a priority.

## Figures and Tables

**Table 1:** Response rates of residents from participating schools*

Province	Response Rate
Newfoundland	26%
Quebec	16%
Ontario	26%
Manitoba	45%
Saskatchewan	11%
Alberta	67%
British Columbia	0%
Overall	34%

* 8 of 10 provinces in Canada have medical schools. Not all schools responded or agreed to participate in the research. No response was received from Nova Scotia.

**Table 2:** Description of sample as a whole and as a function of residency location

Variable	Sample as a Whole (*n* = 141)	Urban (*n* = 112)	Rural (*n* = 29)	*p* value
	Mean (SD)/n (%)*	Mean (SD)/n (%)*	Mean (SD)/n (%)*	
Age	32.13 (5.39)	32.26 (5.48)	31.62 (5.08)	0.57
Gender (Female)	93 (66%)	76 (68%)	17 (59%)	0.35
Years Driving	13.73 (5.32)	14.05 (5.24)	12.48 (5.55)	0.16
Kms Driven**	25,613 (19,436)	22,528 (18,079)	37,103 (20,305)	< 0.001
Kms Driven for Residency Purposes**	16,010 (15,774)	12,663 (12,473)	28,804 (20,252)	< 0.001
**Motor Vehicle Crashes (MVCs)**				
# Residents in MVCs	31 (22%)	20 (17.9%)	11 (37.9%)	0.03
Total # of MVCs	39	26 (67%)	13 (33%)	
# MVCs Per Resident	0.28 (0.61)	0.23 (0.58)	0.45 (0.69)	0.09
***Time of MVC (# of MVCs)***				
Before Work	6 (15%)	4 (15%)	2 (15%)	0.60†
During Work	4 (10%)	4 (15%)	0 (--)	-- †
After Work	14 (36%)	9 (35%)	5 (38%)	0.27†
Unrelated to Work	15 (38%)	9 (35%)	6 (46%)	0.08†
Injury Due to MVC	2 (5%)	1 (4%)	1 (8%)	--
Injury Requiring Medical Attention	1 (3%)	0 (--)	1 (8%)	--
**Close Calls**				
# Residents Involved in Close Calls	76 (54%)	57 (50.9%)	19 (65.5%)	0.21
Total # of Close Calls	206	157 (76%)	49 (24%)	
# Close Calls Per Resident	1.46 (1.72)	1.40 (1.75)	1.69 (1.58)	0.42
***Time of Close Call (# of Close Calls)***				
Before Work	29 (14%)	23 (15%)	6 (12%)	0.79‡
During Work	13 (6%)	9 (6%)	4 (8%)	0.27‡
After Work	111 (54%)	94 (60%)	17 (35%)	0.83‡
Unrelated to Work	53 (26%)	31 (20%)	22 (45%)	0.01‡
**Adverse Motor Vehicle Events (AMVEs)**§				
# Residents Involved in AMVEs	89 (63%)	67 (60%)	22 (76%)	0.08
Total # of AMVEs	245	183 (75%)	62 (25%)	
# AMVEs Per Resident	1.74 (1.87)	1.63 (1.87)	2.14 (1.83)	0.20
**Reason for AMVE**||				
Fatigue	56 (34%)	46 (40%)	10 (21%)	0.67
Driver Inattention	41 (25%)	31 (27%)	10 (21%)	0.50
Weather	39 (24%)	28 (24%)	11 (23%)	0.17
Wildlife	9 (5%)	0 (--)	9 (19%)	< 0.001
Road Conditions	19 (12%)	11 (9%)	8 (17%)	0.03
**Citations**				
# Residents Receiving Citations	53 (38%)	40 (36%)	13 (45%)	0.39

* Mean(SD) is reported for continuous variables (age, etc.); n (%) for categorical variables (gender, etc.).

** Kilometres driven during the first 22 months of residency.

† The data were converted to create two groups (residents with no crash and residents with at least one crash) for rural and urban residents, with associations tested using chi-square test.

‡ The data were converted to create two groups (residents with no close call and residents with at least one close call) for rural and urban residents, with associations tested using chi-square test.

§ Adverse motor vehicle events defined as a crash or a close call.

|| Data presented represent the number of times each reason was cited. Not all respondents provided a reason for their AMVE. Some provided multiple reasons for a single event.

**Table 3:** MVCs and close calls based on kilometres driven* for the sample as a whole and as a function of residency location

Variable	Sample as a Whole (*n* = 141)	Urban (*n* = 112)	Rural (*n* = 29)	*p* value	*Cohen’s d*
	*Mean (SD)*	*Mean (SD)*	*Mean (SD)*		
**Motor Vehicle Crashes (MVCs)**					
# Crashes/100,000 Km Driven	1.35 (3.51)	1.18 (3.33)	1.99 (4.13)	0.27	0.23
# Work Related Crashes/100,000 Km Driven for Residency Purposes	1.35 (4.35)	1.22 (4.29)	1.87 (4.60)	0.47	0.15
**Close Calls**					
# Close Calls/10,000 Km Driven	2.99 (17.66)	3.62 (19.78)	0.56 (0.73)	0.41	0.17
# Work Related Close Calls/10,000 Km Driven for Residency Purposes	2.29 (9.52)	2.74 (10.64)	0.62 (1.86)	0.29	0.22

* Kilometres driven during the first 22 months of residency

**Table 4:** Presence of a driving policy for residents by Canadian university as of August, 2011

University Medical School	Policy
U of British Columbia	No policy in place
U of Alberta	Policy in place
U of Calgary	Policy in place
U of Saskatchewan	No official written policy
U of Manitoba	Unknown*
U of Northern Ontario	Policy in place
U of Western Ontario	No policy in place
McMaster University	Policy in place
U of Toronto	No policy in place
Queens University	Policy in place
University of Ottawa	No policy in place
McGill University	No policy in place
Dalhousie University	Unknown*
Memorial University	No policy in place

* University did not respond to requests for information.

## Appendix A The University of Alberta Travel Policy for Residents

### TRAVEL POLICY FOR RURAL ALBERTA NORTH

For all residents who are scheduled into rural family medicine block rotations there is an implication of travel with respect to attending academic programming. Obviously, the further a resident is stationed from their home base the wider the implications with respect to that travel.

DISTANCE:

Traveling long distances to attend academic programming can take the learner away from the rotation for inordinate amounts of time to account for that travel. Academic programming will be scheduled in a way to minimize time away from rotation related to travel (ie, Fridays or Mondays). A resident who is stationed within 350 km of one of the home bases will be expected to attend academic programs including the monthly academic day, at the closest home base. A resident who is stationed greater than 400 km away from one of the home bases will not be expected to attend the monthly academic day but will be expected to make the effort to attend specific workshops or academic courses provided by the program. In lieu of not attending the monthly academic day, every effort should be made to attend the didactic sessions via video-conferencing, as this portal is already set up between Red Deer and Grande Prairie. Residents who are stationed in a location between 350 km and 400 km can view their attendance to the monthly academic day as discretionary but need to discuss this ahead of time with their preceptor and then the Co-Director.

ROAD SAFETY:

Road conditions are not always safe to travel. Resident safety must be given priority. Thus if poor road conditions compromise a resident’s safety, that individual will be excused from attending mandatory programming. Non attendance because of poor road conditions will need to be discussed with the preceptor and the site coordinator at the time. When possible, video conferencing will be arranged to allow the resident to participate in that way.

The program is exploring other ways for learner’s to participate in programming remotely.

SITES CLOSE TO HOME BASE:

In the Department of Family medicine there has been a longstanding policy not to reimburse travel to community teaching sites that are close to the learner’s home base. This includes all sites that are within 50 km. Rural Alberta North will continue to uphold this policy and will not reimburse for travel to community teaching sites less than 50 km from either Grande Prairie or Red Deer (as per the RPAP mileage chart). For sites that are more than 50 km distant and do not have full time accommodation available, a “commuting expense” will be reimbursed at a flat rate of $100/week. For such a site, it is recognized that suitable accommodation needs to be provided for the resident when on-call. When full time accommodation is available and the learner decides not to take advantage of this provision, there will be no reimbursement for travel as it is recognized that commuting is then the learner’s own responsibility.
